# Endoscopic-guided chemoablation of an inoperable main-duct intraductal papillary mucinous neoplasm

**DOI:** 10.1093/jscr/rjag820

**Published:** 2026-09-21

**Authors:** Adam Bolad, Dhruv J Patel, Thoa Tran, John M DeWitt, C Max Schmidt

**Affiliations:** Tulane University School of Medicine, New Orleans, LA, United States; Department of Surgery, Indiana University School of Medicine, Indianapolis, IN, United States; Department of Surgery, Indiana University School of Medicine, Indianapolis, IN, United States; Department of Medicine, Indiana University School of Medicine, Indianapolis, IN, United States; Department of Surgery, Indiana University School of Medicine, Indianapolis, IN, United States

**Keywords:** IPMN, chemoablation, pancreatic cysts, mesenteritis

## Abstract

Sclerosing mesenteritis (SM) is a rare inflammatory condition causing thickening/foreshortening of the mesentery, challenging the performance of pancreatic surgery. We report a patient with main-duct intraductal papillary mucinous neoplasm (IPMN) who had concomitant SM for whom operative management was not feasible. After discussion with gastroenterology, an alternative treatment approach was employed. The patient is a 58-year-old male who presented with main-duct IPMN of the pancreatic head with main pancreatic duct dilation >10 mm. At operative exploration, he was found to have SM, preventing the ability to perform a tension-free reconstruction as part of a pancreatoduodenectomy. The patient was instead offered interval sessions of endoscopic ultrasound guided (EUS) guided chemoablation with gemcitabine/paclitaxel under compassionate use. Surveillance cytology and molecular testing*,* initially notable for a guanine nucleotide-binding protein, alpha-stimulating activity polypeptide (*GNAS*) gene mutation, has been negative for 3 years. The patient remains asymptomatic without adverse events. This case demonstrates the potential application of EUS-guided chemoablation for select inoperable IPMN.

## Introduction

Current management of intraductal papillary mucinous neoplasm (IPMN) is guided by the recent 2024 Kyoto Guidelines, stratifying lesions based on select criteria [1]. Main pancreatic duct dilation (MPD) ≥10 mm is one high-risk stigmata (HRS) of IPMN, associated with a 40% chance of progression to invasive cancer [2]. Surgical resection is the standard of management for IPMN with HRS. Yet, sclerosing mesenteritis (SM), a rare fibro-inflammatory condition of the abdominal mesentery, poses a technical barrier to pancreatic surgery [3].

Pharmacologic ablation under endoscopic ultrasound (EUS) guidance is a therapeutic alternative to surgical resection of pancreatic cysts, though literature for main-duct IPMN is sparse [4]. Across prospective trials, EUS-guided pancreatic cyst ablation has achieved meaningful response rates: 35% with ethanol alone, 50%–79% when paclitaxel is added to ethanol lavage, and 61%–67% with alcohol free gemcitabine-paclitaxel protocols [5]. Herein we report the case of a patient harboring an IPMN with HRS of main duct dilation >10 mm whose operative candidacy was precluded by SM. He was subsequently managed with serial EUS-guided chemoablation, demonstrating sustained radiographic and molecular stability.

## Case report

Our patient is a 58-year-old male with a history of recurrent acute pancreatitis secondary to pancreatic divisum. The patient presented in 2008 to the gastroenterology services at our institution and was managed initially with minor papilla sphincterotomy with stent placement. Cross-sectional imaging at that time was notable for SM. He was subsequently followed by gastroenterology for recurrent episodes of pancreatitis as well as surveillance of the SM. In 2014, he was found on imaging to have MPD dilation (7 mm in pancreatic head) with suspected duct stricture just proximal to the ampulla of Vater. Although offered surgical referral, he elected for endoscopic management. After multiple stent exchanges, there was persistent MPD dilation (up to 5.5 mm) in 2017, which progressively increased to 12 mm in March 2019. Biopsy of the minor papilla at that time revealed preserved villous architecture and was negative for dysplasia or malignancy. Duct fluid analysis was notable for an elevated carcinoembryonic antigen (CEA) (885.7 ng/ml), with molecular testing positive for a GNAS mutation. The patient was subsequently provided a referral to the Indiana University Health Pancreatic Cyst and Cancer Early Detection Clinic for surgical management of suspected main duct IPMN.

**Figure 1:**
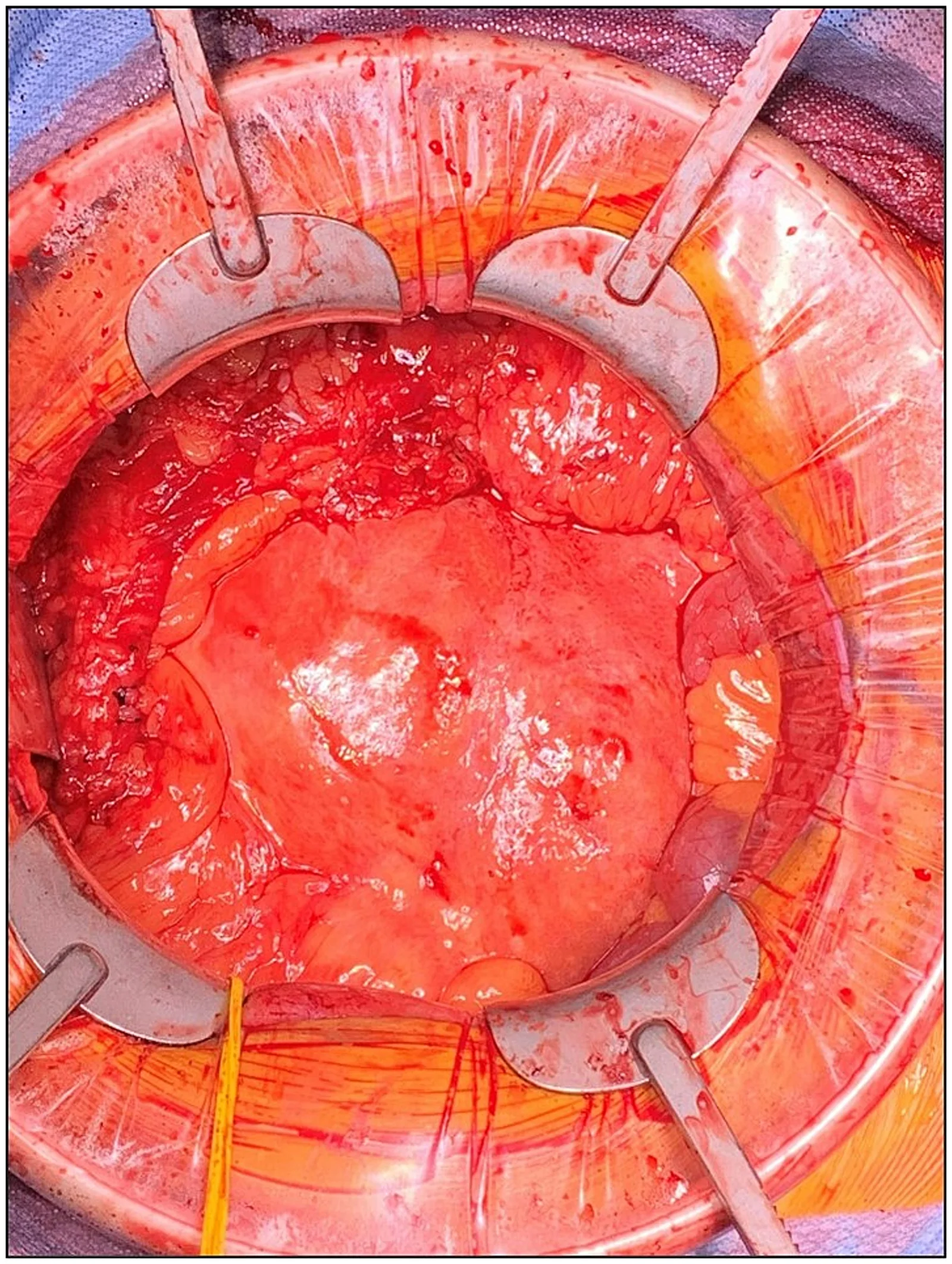
Intraoperative image of the patient’s inflamed mesentery taken at time of initial operative exploration in June 2019.

**Figure 2:**
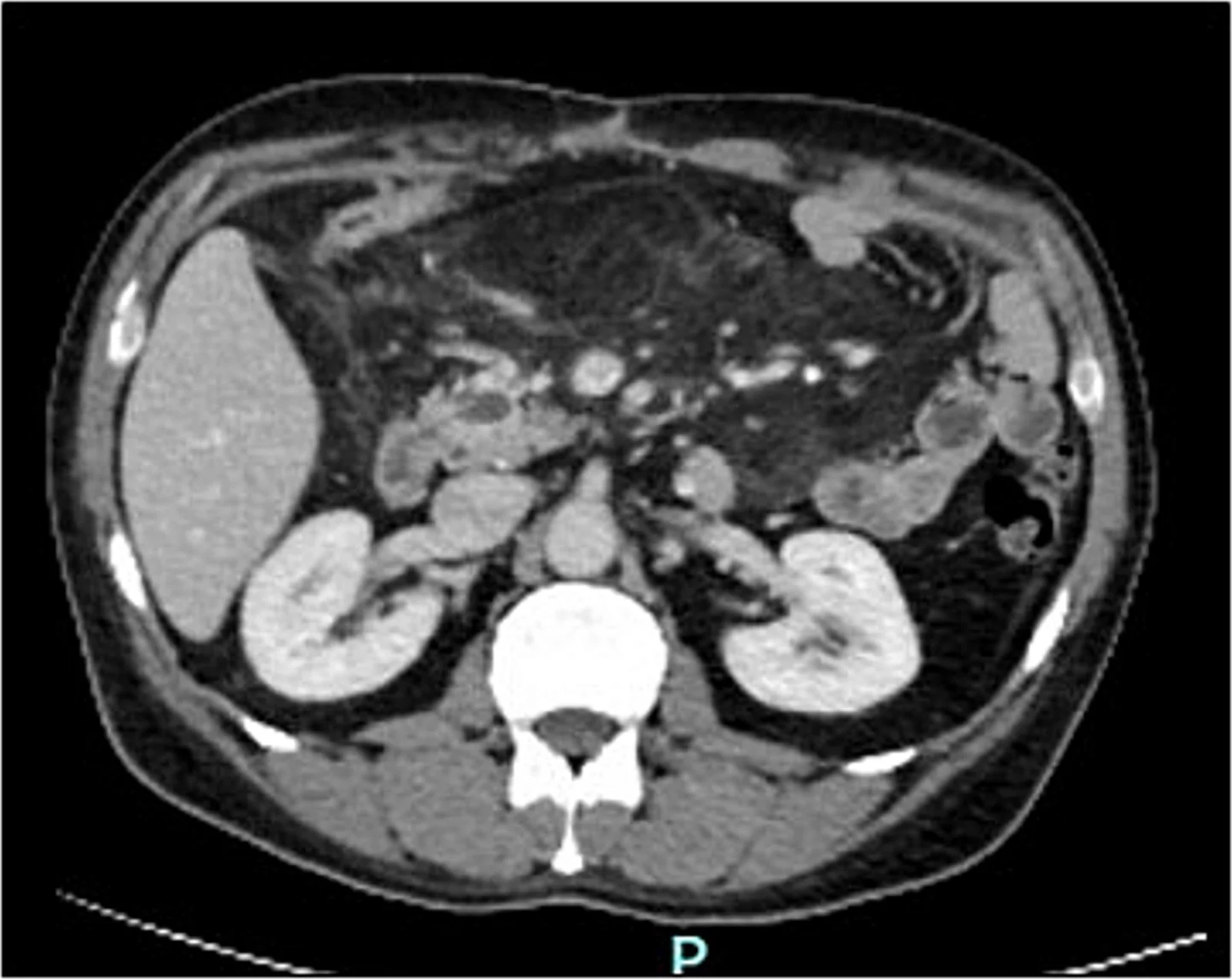
Computed tomography imaging of the patient in January 2020 demonstrating persistent mesenteric haziness compatible with ongoing sclerosing mesenteritis.

The patient was consented for pancreatoduodenectomy following evaluation in our clinic in May 2019. Intraoperative evaluation was notable for extensive mesenteric inflammation and foreshortening of the mesentery (Fig. 1). A tension-free reconstruction was not feasible, and the resection was aborted. A trial of corticosteroids was ineffective based on imaging in January 2020 (Fig. 2). Since the patient was asymptomatic, he was followed with imaging surveillance. In the setting of persistent MPD dilation, a second operative exploration was performed in February 2022. Intraoperative findings were notable for worsening mesenteric fibrosis, and the procedure was again aborted. Following multidisciplinary discussion the patient was referred back to gastroenterology for pancreatic ductoscopy in order to determine whether the MPD dilation was related more to pancreatitis or IPMN.

**Figure 3:**
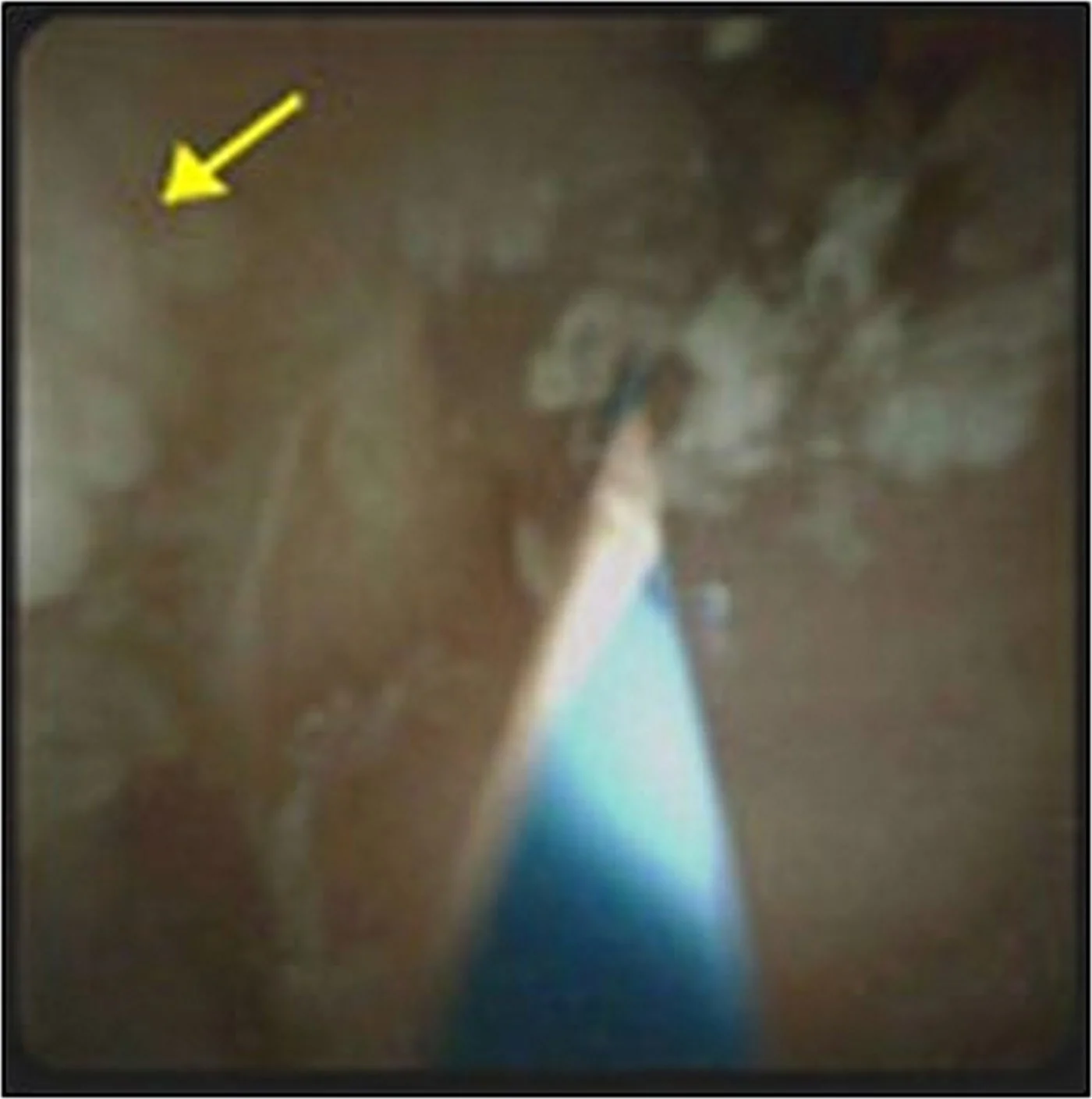
Pancreatic ductoscopy in March 2022 revealing frond-like projections (indicated by yellow arrow) in the pancreatic duct consistent with diagnosis of main-duct IPMN.

**Figure 4:**
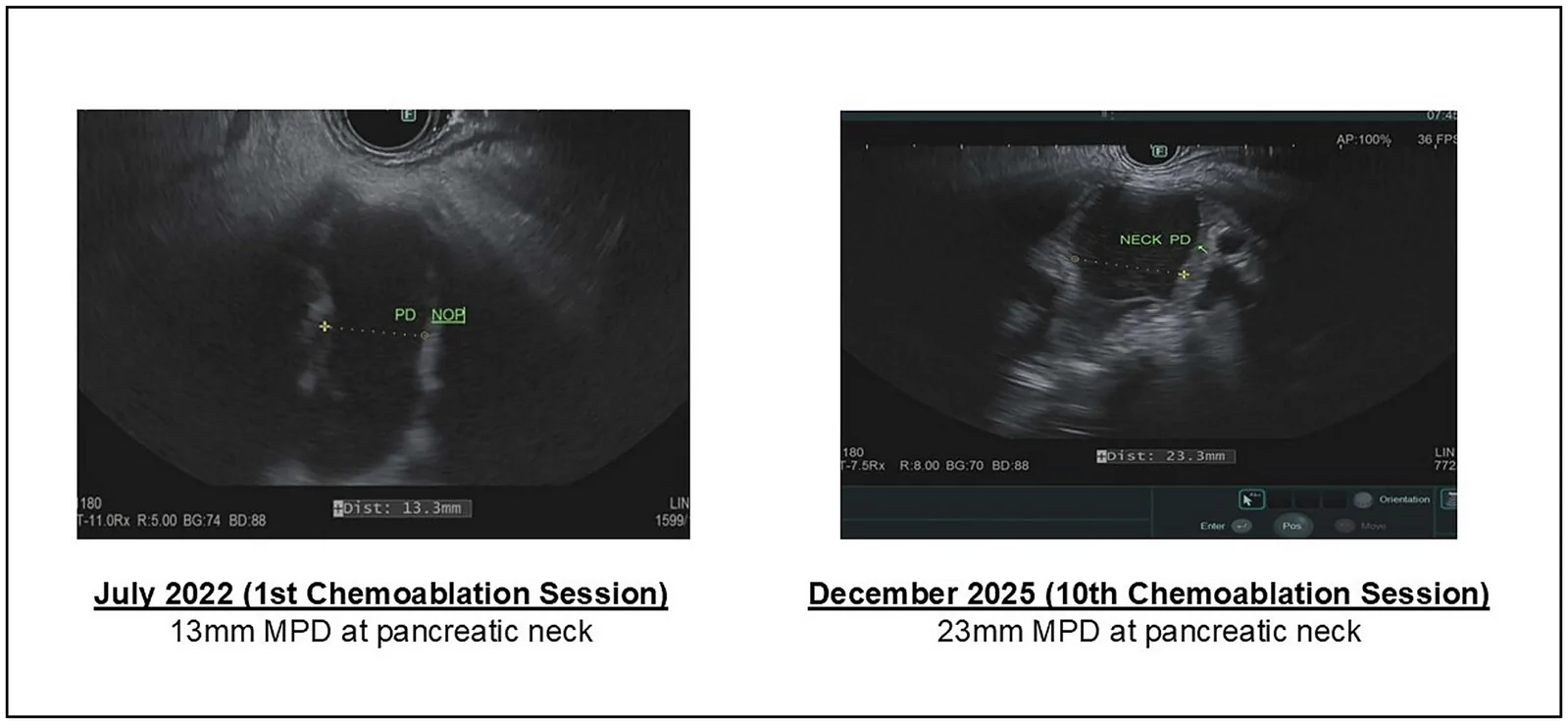
Endoscopic ultrasound imaging of the patient’s main pancreatic duct at the level of the pancreatic neck.

Ductoscopy revealed mucin and frond-like projections, consistent with diagnosis of IPMN (Fig. 3). Biopsies of the duct revealed columnar epithelium with mucinous cytoplasm and low-grade dysplasia. There was no evidence of high-grade dysplasia or malignancy. As options for definitive management were limited in the setting of unreconstructible disease, multivisceral transplant, and EUS-guided chemoablation under compassionate use were considered. The latter option was selected. The first session took place in July 2022 and consisted of a 25 cc instillation of gemcitabine (19 mg/cc) and paclitaxel (3 mg/cc). No adverse advents were observed. Initially, sessions occurred every 3 months and have since been extended out to 6 months. To evaluate treatment response, interval cytology was obtained as well repeat molecular testing, along with serum tumor markers. Duct fluid analysis in March 2023 revealed no dysplasia. Molecular testing performed in May 2025 returned negative for any mutations. Serum CEA and carbohydrate antigen 19-9 (CA 19-9) have remained non-elevated. While the MPD continues to increase in diameter (from 13 mm in July 2022 to 23 mm in December 2025), the most recent duct cytology in December 2025 revealed no atypical cells or high-grade dysplasia or malignancy (Fig. 4). The patient remains asymptomatic and continues to tolerate serial endoscopic chemoablation.

## Discussion

This case highlights the complexity in the management of a patient harboring an IPMN with high-risk features for malignancy, who also possessed a rare inflammatory condition of the mesentery, SM. Case studies and reviews suggest that SM is detected incidentally in <1% of abdominal imaging scans [6]. Pancreatic resection in the setting of SM is often unfeasible due to consequent encasement of the mesenteric vasculature and dense fibrosis [7]. In a study of six patients with SM involving the pancreas, five were initially suspected of harboring primary pancreatic neoplasms with several undergoing exploratory laparotomy before a diagnosis of benign was assigned [8]. Medical management of SM with corticosteroids, tamoxifen, and azathioprine produces variable response rates [9, 10]. There is no consensus treatment for SM.

EUS-guided ablation of pancreatic cysts is a minimally invasive modality for patients who are poor surgical candidates or decline surgery [11]. Studies from our institution show that EUS-guided ethanol lavage followed by paclitaxel instillation achieves near complete radiological resolution in a substantial portion of mucinous cysts and branch duct IPMNs [5, 12]. While a multivisceral transplant (MVT) was considered as a definitive surgical option for the patient in the present case, the morbidity of MVT along with the complexity of delivering chemotherapy with postoperative immunosuppression (in the setting of potential invasive carcinoma on surgical pathology) hindered the feasibility of a MVT. MPD dilation >5 mm has been listed as a relative contradiction to ablation in previous prospective trials; therefore, the present case highlights a novel investigational application of ablative approaches and an expansion of potential indications [5]. EUS-guided cyst ablation with ethanol and paclitaxel has also been shown to eliminate the detectable oncogenic mutation *GNAS* in cyst fluid following treatment, providing molecular evidence of treatment response [13]. This finding is relevant to our patient as he demonstrated interval clearance of a *GNAS* mutation on serial molecular testing following chemoablation. Additionally, our patient’s MPD continues to increase in size over time. In the absence of development of high-grade dysplasia or invasive carcinoma, it is unclear whether this size increase represents disease progression versus changes secondary to chemoablation. It is possible that the instillation of chemotherapy in the cyst cavity is causing physical dilation of the cyst as well as ductal irritation. Future ductoscopy will help assist with identification of treatment response and determination of treatment duration. This is in contrast to a prior case reported by Hartz *et al*. in which decreasing MPD diameter was observed following endoscopic treatment of a main-duct IPMN [14].

We acknowledge multiple limitations of the present case. While we present 3 years of follow-up after initiation of chemoablation, there is limited follow-up overall to assess for the development and timing of malignant transformation. The absence of surgical pathology, while inherent to our patient’s inoperable disease secondary to SM, limits us from definitively excluding occult invasive carcinoma.

This case highlights how a benign fibro-inflammatory mesenteric process can preclude standard pancreatic resection for IPMN. There is a role for multidisciplinary management and consideration of novel investigational strategies, such as endoscopic chemoablation. While not equivalent to standard surgical extirpation of main-duct IPMN with HRS, the favorable long-term stability observed in our patient suggests that chemoablation may offer a pragmatic compromise between oncologic control and procedural risk.
